# Distribution of Pathogens in Elderly Chinese Patients With Pneumonia: A Systematic Review and Meta-Analysis

**DOI:** 10.3389/fmed.2021.584066

**Published:** 2021-07-26

**Authors:** Luming Chen, Hongqiang Huang, Xiaolin Chen

**Affiliations:** Department of Geriatrics, Guangdong Provincial Hospital of Traditional Chinese Medicine, Guangzhou, China

**Keywords:** antibiotics, distribution of pathogens, elderly patients, pneumonia, clinical application

## Abstract

**Background:** To summarize the distribution of pathogenic bacteria in elderly Chinese patients with pneumonia and provide guidance for the clinical application of antibiotics.

**Methods:** The electronic databases of PubMed, Embase, Cochrane library, and China National Knowledge Infrastructure were searched. The primary outcomes included the prevalence of gram-positive cocci, gram-negative bacilli, and fungus. The summary prevalence and 95% confidence interval (CI) were calculated using the random-effects model.

**Results:** A total of 17 retrospective studies reporting a total of 5,729 elderly patients with pneumonia were selected for final analysis. The summary prevalence of gram-positive cocci was 25% (95% CI: 20–30%; *p* < 0.001), whereas the prevalence of gram-negative bacilli was 56% (95% CI: 46–67%; *p* < 0.001). Moreover, the pooled prevalence of fungus in elderly patients with pneumonia was 11% (95% CI: 8–14%; *p* < 0.001). The most common gram-positive cocci were *Staphylococcus aureus* (ES: 8%; 95% CI: 6–11%; *p* <0.001), *Streptococcus hemolyticus* (ES: 7%; 95% CI: 6–8%; *p* < 0.001), and *Streptococcus pneumoniae* (ES: 5%; 95% CI: 3–7%; *p* < 0.001). *Pseudomonas aeruginosa* (ES: 18%; 95% CI: 14–22%; *p* <0.001) and *Klebsiella pneumoniae* (ES: 14%; 95% CI: 11–18%; *p* <0.001) were most common gram-negative bacilli. Furthermore, the pooled prevalence of *Candida albicans* in elderly patients with pneumonia was 6% (95% CI: 5–8%; *p* < 0.001).

**Conclusions:** The findings demonstrated the comprehensive distribution of pathogenic bacteria in elderly Chinese patients with pneumonia, which could guide further antibiotic therapies.

## Introduction

Pneumonia is the leading cause of infection-related deaths worldwide and the fourth-highest all-cause mortality in elderly patients (older than 65 years). It is characterized by cough, sputum production, dyspnea, and chest pain (1, 2). Underlying comorbid diseases, impaired mucociliary clearance, and waning immunity have been identified as risk factors for the incidence of pneumonia in elderly patients. The annual incidence of pneumonia in the elderly is nearly four times that of younger populations (3). The number of elderly patients with pneumonia is rapidly increasing due to increasing sociodemographic aging, which has become a global problem. Moreover, the incidence of hospitalization due to pneumonia has significantly increased, and the burden of community-acquired pneumonia is more significant due to an expected 20% of the global population reaching elderly status by 2050 (4, 5).

Recently, the number of elderly patients with pneumonia has significantly increased in China due to the gradual increase in the aging population. Moreover, severe pneumonia was the main cause of death in elderly patients. Effective treatment strategies should be given to elderly patients with pneumonia to improve the prognosis through early diagnosis and treatment. Although there is the widespread use of vaccines and antibiotics, the prognosis for pneumonia in elderly individuals remains poor, and the pathogens were not systematically analyzed. Therefore, the current meta-analysis was conducted to illustrate the distribution of pathogenic bacteria in elderly Chinese patients with pneumonia, guiding the specific treatment strategies for such patients.

## Methods

### Data Sources, Search Strategy, and Selection Criteria

This review was conducted and reported according to the Preferred Reporting Items for Systematic Reviews and Meta-Analysis Statement issued in 2009 (6). Any study investigating the distribution of pathogenic bacteria in elderly Chinese patients with pneumonia was eligible, and no restrictions were placed on publication status and language. Electronic searches of the PubMed, Embase, Cochrane library, and China National Knowledge Infrastructure databases were conducted for articles published in June 2019. The core search terms included “senile pneumonia” OR “elderly pneumonia” AND “pathogenic bacteria.” The reference lists of retrieved studies were also reviewed to identify any new eligible studies.

Two authors independently evaluated and screened the potential studies. Any disagreement between these two authors was settled by group discussion or adjudicated by an additional author when necessary. The inclusion criteria for studies were as follows: (1) all participants diagnosed with pneumonia and aged ≥60 years; (2) patients received sputum culture analysis; (3) the study at least reported one of the prevalence of gram-positive cocci, gram-negative bacilli, and fungus. Moreover, the distribution of specific types of pathogenic bacteria was also summarized; and (4) prospective or retrospective study design.

### Data Collection and Quality Assessment

Data from the included studies were independently abstracted and crosschecked by two authors using a standardized data extraction form, and any disagreement was settled by group discussion until a consensus was reached. The collected items included the first author's last name, publication year, study period, region, study design, sample size, age range, number of men and women, pneumonia subtypes, pathogen analysis, and the distribution of pathogenic bacteria. The quality of included studies was assessed by the Newcastle–Ottawa Scale, which is based on selection (four items: 4 stars), comparability (one item: 2 stars), and outcome (three items: 3 stars) (7). The “star system” for assessment of retrieved studies ranged from 0 to 9. Two authors independently evaluated the quality of included studies, and any disagreement was adjudicated by an additional author after referring to the original article.

### Statistical Analysis

The prevalence (cases/patients) of gram-positive cocci, gram-negative bacilli, and fungus and the distribution of specific types of pathogenic bacteria were assigned as event and total sample size in each study. After that, the summary prevalence for investigated outcomes was calculated using the random-effects model (8, 9). The heterogeneity across included studies was assessed using I-square and Q statistic, and I-square > 50.0% or *p* < 0.10 were considered as significant heterogeneity (10). Sensitivity analyses were conducted for gram-positive cocci, gram-negative bacilli, and fungus to assess the influence of every single study. Subgroup analyses for the prevalence of gram-positive cocci, gram-negative bacilli, and fungus were calculated based on mean age, percentage male, and study quality. Publication biases for investigated outcomes were evaluated using the funnel plots and Egger and Begg tests (11, 12). Moreover, the trim and fill method was used to adjust potential publication bias if significant publication bias was detected (13). All reported *p*-values are two-sided, and *p* < 0.05 was considered statistically significant for all included studies. Statistical analyses were performed using STATA software (version 10.0; Stata Corporation, College Station, TX, USA).

## Results

### Literature Search

A total of 463 studies were identified in the initial search of the databases based on the search strategy mentioned earlier, of which 121 were excluded due to duplicate topics. An additional 317 studies were excluded because these were other types of articles (i.e., case reports, review articles, scientific abstracts) and studies not relevant to our study. The remaining 25 studies were retrieved for further evaluations, of which eight studies were excluded due to the following reasons: intervention study (*n* = 4), drug resistance study (*n* = 3), and review (*n* = 1). A total of 17 studies were selected for final analysis, and manual searching of the reference lists did not identify any new eligible study (14–30). The study selection process is presented in Supplementary Figure 1.

### Study Characteristics

The 17 identified studies had a retrospective study design and included 5,729 elderly patients with pneumonia. The baseline characteristics of included studies and patients are summarized in Supplementary Table 1. The publication year ranged from 1996 to 2008, and 89–1,636 patients were included in each trial. The study period ranged from 1992 to 2016, and all patients received sputum culture analysis. Five studies included patients presented with community-acquired pneumonia and hospital-acquired pneumonia, one study contained patients with community-acquired pneumonia, whereas the remaining 11 studies did not mention the pneumonia subtypes. All studies were published in Chinese, and the quality of included studies was low. The quality of included studies was assessed using the Newcastle–Ottawa Scale, and a study with 7–9 stars was regarded as high quality. Of the 17 included studies, six studies got 5 stars, nine studies with 4 stars, and the remaining two studies with 3 stars.

### Gram-Positive Cocci

**Figure 1:**
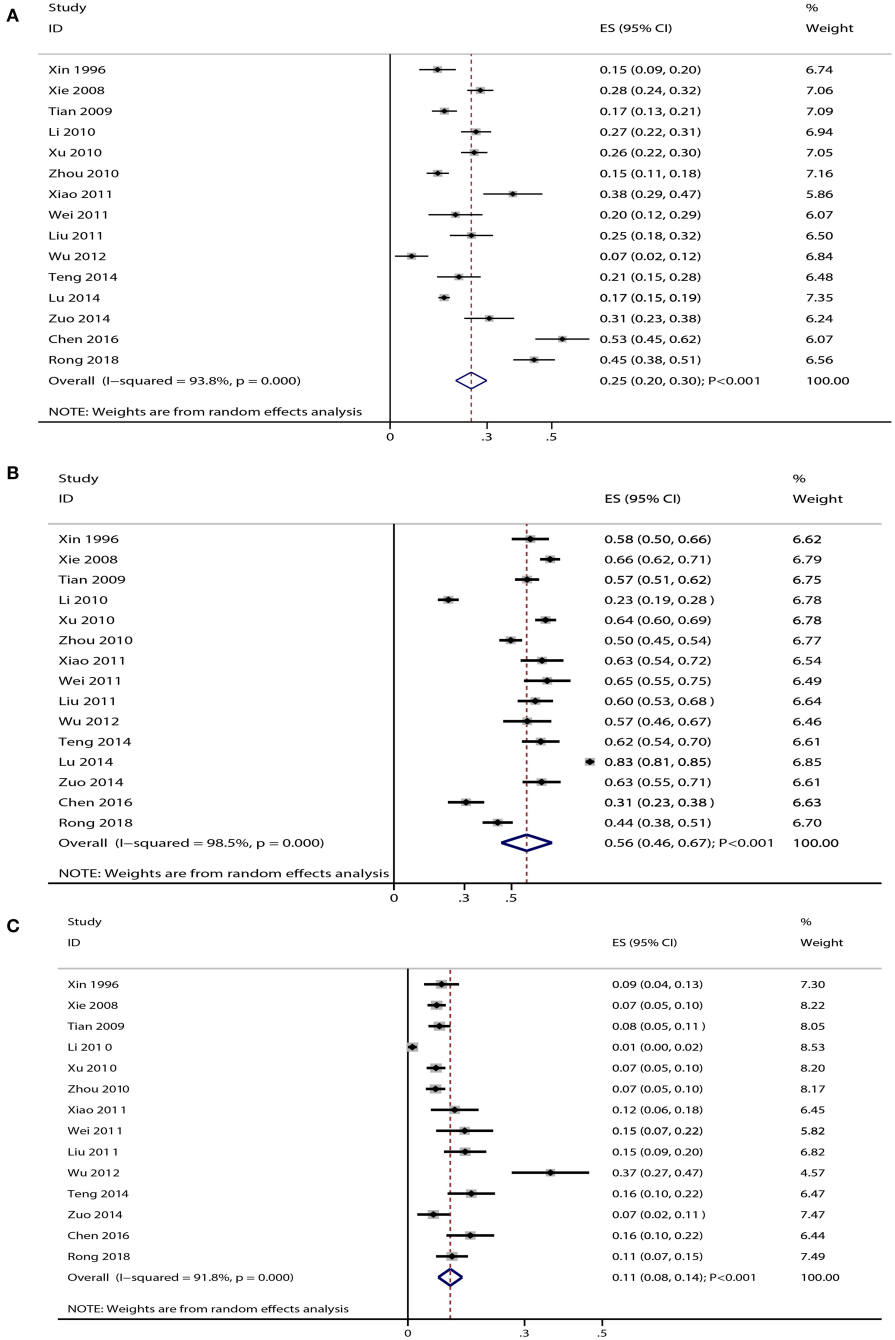
**(A)** Summary prevalence for gram-positive cocci in elderly patients with pneumonia. **(B)** Summary prevalence for gram-negative bacilli in elderly patients with pneumonia. **(C)** Summary prevalence for fungus in elderly patients with pneumonia.

Data for the distribution of gram-positive cocci were available in 15 studies, and the summary prevalence of gram-positive cocci was 25% (95% CI: 20–30%; *p* < 0.001; Figure 1A). Moreover, substantial heterogeneity was detected among the included studies (I-square: 93.8%; *p* < 0.001). Sensitivity analysis indicated that the prevalence of gram-positive cocci ranged from 19 to 31% by sequentially excluding every individual study (Supplementary Figure 2). Moreover, potential significant publication bias for gram-positive cocci was detected (*p*-value for Egger: 0.030; *p*-value for Begg: 0.092; Supplementary Figure 3), and the prevalence of gram-positive cocci was 29% after adjustment using the trim and fill method (95% CI: 23–35%; *p* < 0.001; Supplementary Figure 4).

### Gram-Negative Bacilli

Data for the distribution of gram-negative bacilli were available in 15 studies, and the pooled prevalence of gram-negative bacilli was 56% (95% CI: 46–67%; *p* < 0.001; Figure 1B). There was significant heterogeneity among the included studies (I-square: 98.5%; *p* < 0.001). Sensitivity analysis indicated that the prevalence of gram-negative bacilli ranged from 44 to 69% by sequentially excluding every individual study (Supplementary Figure 5). The Begg test indicated no significant publication bias for gram-negative bacilli (*p* = 0.553), whereas the Egger test indicated potential significant publication bias (*p* = 0.011) (Supplementary Figure 6). The prevalence of gram-negative bacilli was 67% after adjustment using the trim and fill method (95% CI: 42–90%; *p* < 0.001; Supplementary Figure 7).

### Fungus

Data for the distribution of fungus were available in 14 studies, and the summary prevalence for fungus was 11% (95% CI: 8–14%; *p* < 0.001; Figure 1C). There was no significant heterogeneity among the included studies (I-square: 91.8%; *p* < 0.001). Sensitivity analyses indicated that the prevalence of fungus was 7–15% by sequentially excluding every individual study (Supplementary Figure 8). Moreover, there was significant publication bias for fungus (*p*-value for Egger: <0.001; *p*-value for Begg: 0.006; Supplementary Figure 9), and the prevalence of fungus was 9% after adjustment using the trim and fill method (95% CI: 6–12%; *p* < 0.001; Supplementary Figure 10).

### Specific Type of Pathogenic Bacteria

The summarized results for the prevalence of specific type of pathogenic bacteria are presented in Table 1. The summary prevalence of *Staphylococcus aureus* (ES: 8%; 95% CI: 6–11%; *p* < 0.001), *Streptococcus hemolyticus* (ES: 7%; 95% CI: 6–8%; *p* < 0.001), and *Streptococcus pneumoniae* (ES: 5%; 95% CI: 3–7%; *p* < 0.001) indicated that they were the most common gram-positive cocci. Moreover, the pooled prevalence of *Staphylococcus epidermidis* and *coagulase-negative Staphylococcus* were 4% (95% CI: 3–6%; *p* < 0.001) and 3% (95% CI: 2–4%; *p* < 0.001), respectively. In addition, *Pseudomonas aeruginosa* (ES: 18%; 95% CI: 14–22%; *p* < 0.001) and *Klebsiella pneumoniae* (ES: 14%; 95% CI: 11–18%; *p* < 0.001) were the two most common gram-negative bacilli in elderly patients with pneumonia. Moreover, the prevalence of other specific types of gram-negative bacilli ranged from 1 to 8%. The pooled prevalence of *Candida albicans* in elderly patients with pneumonia was 6% (95% CI: 5–8%; *p* < 0.001), with no evidence of heterogeneity.

**Table 1:** Summary results for specific pathogenic bacteria.

**Pathogenic bacteria**	**Number of studies**	**Prevalence and 95% CI**	***p*****-value**	**Heterogeneity (%)**	***p*****-value for Heterogeneity**	**Egger test**	**Begg test**
*Staphylococcus aureus*	16	0.08 (0.06–0.11)	<0.001	90.1	<0.001	0.017	0.034
*Coagulase-negative staphylococcus*	8	0.03 (0.02–0.04)	<0.001	65.4	0.005	0.005	0.009
*Staphylococcus epidermidis*	9	0.04 (0.03–0.06)	<0.001	79.2	<0.001	0.027	0.048
*Streptococcus pneumoniae*	12	0.05 (0.03–0.07)	<0.001	87.7	<0.001	0.004	0.003
*Streptococcus hemolyticus*	7	0.07 (0.06–0.08)	<0.001	0.0	0.965	0.352	0.368
*Klebsiella pneumonia*	17	0.14 (0.11–0.18)	<0.001	92.4	<0.001	0.200	0.127
*Pseudomonas aeruginosa*	16	0.18 (0.14–0.22)	<0.001	94.1	<0.001	0.026	0.444
*Actinobacter baumannii*	13	0.08 (0.06–0.11)	<0.001	92.1	<0.001	0.300	0.067
*Escherichia coli*	17	0.08 (0.07–0.09)	<0.001	65.7	<0.001	0.074	0.019
*Enterobacter layer*	4	0.07 (0.02–0.11)	0.002	92.3	0.001	0.010	0.308
*Bacillus levans*	13	0.03 (0.02–0.05)	<0.001	86.5	<0.001	0.007	0.200
*Proteus vulgaris*	5	0.02 (0.01–0.04)	0.010	87.6	<0.001	0.050	0.027
*Stenotrophomonas maltophilia*	13	0.04 (0.03–0.05)	<0.001	66.1	<0.001	0.013	0.033
*Acinetobacter lwoffii*	3	0.02 (0.01–0.02)	<0.001	0.0	0.872	0.163	0.296
*Hemophilus parainfluenzae*	6	0.03 (0.01–0.05)	0.001	88.5	<0.001	0.026	0.024
*Citrobacter freundii*	3	0.01 (0.00–0.02)	0.002	0.0	0.913	0.163	0.296
*Pseudomonas alcaligenes*	4	0.01 (0.00–0.02)	0.040	55.9	0.079	0.125	0.089
*Candida albicans*	8	0.06 (0.05–0.08)	<0.001	0.0	0.880	0.482	0.386

### Subgroup Analyses

Subgroup analyses for the prevalence of gram-positive cocci, gram-negative bacilli, and fungus based on mean age, percentage male, and study quality were conducted (Supplementary Table 2). The prevalence of gram-positive cocci was high if the mean age was >75 years or the study was low quality. Moreover, patients aged ≥75 years, percentage male >70.0%, and study with high quality were associated with a high prevalence of gram-negative bacilli. The prevalence of fungus was high if the mean age of patients was >75 years, percentage male <70.0%, or study was low quality.

## Discussion

Pneumonia is the most common respiratory disease, and antibiotics are widely used for treating patients diagnosed with pneumonia. The incidence of pneumonia in the elderly is high due to organ function decline, cough reflex, and decrease in swallowing ability and bronchial mucociliary clearance. However, the data on the distribution of pathogenic bacteria in elderly Chinese patients with pneumonia are limited and inconclusive. The current quantitative meta-analysis recruited 5,729 elderly patients with pneumonia from 17 retrospective studies, with a wide range of patient characteristics. The findings of this study systematically reported the prevalence of gram-positive cocci, gram-negative bacilli, and fungus in elderly Chinese patients with pneumonia. Moreover, the prevalence of the specific type of pathogenic bacteria was illustrated. Furthermore, whether the prevalence of gram-positive cocci, gram-negative bacilli, and fungus are different according to mean age, percentage male, and study quality were assessed. The results of this study could guide the use of antimicrobial agents in elderly patients with pneumonia.

The current study indicated that the prevalence of gram-positive cocci in elderly patients with pneumonia was 25% (95% CI: 20–30%; *p* < 0.001), and the most common gram-positive cocci were *S. aureus, S. hemolyticus*, and *S. pneumoniae*. Xie et al. reported that the susceptibility rate of vancomycin was 100% for patients infected by gram-positive cocci, whereas the susceptibility to cefazolin sodium and ampicillin sodium was lower (15, 18). Moreover, Teng et al. suggested that gram-positive cocci are sensitive to vancomycin and teicoplanin (26). Therefore, the sensitivity of gram-positive cocci to cephalosporins, penicillin, quinolones, and trimethoprim was low, whereas the sensitivity to vancomycin and teicoplanin was higher. The prevalence of gram-negative bacilli in elderly patients with pneumonia was 56% (95% CI: 46–67%; *p* < 0.001), and the most common gram-negative bacilli were *P. aeruginosa* and *K. pneumoniae*. Tian et al. reported that the resistance to ampicillin was highest, whereas resistance to imipenem was lowest, with the resistance rate from 0 to 14.3% for gram-negative bacilli (16). Xu et al. noted that the susceptibility rate of gram-negative bacilli to imipenem/cilastatin sodium reached 91% (18). The sensitivity of gram-negative bacilli to quinolones (ciprofloxacin, ofloxacin, ceftriaxone, and ceftazidime) and the third generation of cephalosporins was low, whereas the sensitivity to imipenem/cilastatin was high. The potential reasons for this could be: (1) quinolones are widely used as antimicrobial agents in China, and the pathogenic bacteria have high resistance to quinolones; (2) *P. aeruginosa, K. pneumoniae*, and other gram-negative bacilli could still induce gene mutation in beta-lactamase after treatment with the third generation of cephalosporins. Hence, it is necessary to formulate ultra-broad-spectrum beta-lactamase, which will be associated with a reduction in pneumonia pathogen susceptibility to antimicrobial agents. Therefore, the imipenem/cilastatin should be used for gram-negative bacilli owing to these antibiotics did not cross-resistance with other beta-lactamases (28). The prevalence of fungus in elderly patients with pneumonia was 11% (95% CI: 8–14%; *p* < 0.001), and the most common fungus was *C. albicans*. The potential reason for this could be because most elderly patients with lower respiratory tract infections have low resistance and comorbidity with other serious diseases. Moreover, the widespread use of broad-spectrum antibiotics and immunosuppressive agents causes susceptibility in patients. Furthermore, the *C. albicans* was contamination of upper airway secretion but not a pathogen for pneumonia. Patients presented positive for *C. albicans* could be caused by other pathogens. Therefore, an effective strategy should be used to prevent the spread of fungal infections.

Sensitivity analyses in the current study indicated the influence of a single study from the overall prevalence of gram-positive cocci, gram-negative bacilli, and fungus in elderly patients with pneumonia. The pooled prevalence for gram-positive cocci ranged from 20 to 30%, and the 95% CI for the prevalence of gram-positive cocci ranged from 19 to 31% by sequentially excluding every individual study, which indicated that the prevalence for gram-positive cocci was stable. Moreover, the summary prevalence for gram-negative bacilli ranged from 46 to 67%, and after sequentially excluding each study, the 95% CI for the prevalence of gram-negative bacilli ranged from 44 to 69%. The potential reason for this change could be the study conducted by Lu et al. (27), which specifically included elderly patients in an island area. The pooled prevalence for fungus in elderly patients with pneumonia ranged from 8 to 14%. The result of sensitivity analysis indicated that after sequentially excluding every individual study, the prevalence of fungus ranged from 7 to 15%, which indicated that the pooled prevalence of fungus in elderly patients with pneumonia had relatively high stability.

Subgroup analyses indicated that older patients could be easily infected with gram-positive cocci, gram-negative bacilli, and fungus, which might be the cause of the high risk of pneumonia in elderly patients. Moreover, percentage male >70.0% showed a relatively high prevalence of gram-negative bacilli, whereas the prevalence of fungus was relatively high when percentage male was <70.0%. These results suggested that males could be easily infected with gram-negative bacilli, whereas females had a relatively high prevalence of fungal infection. The prevalence of gram-positive cocci, gram-negative bacilli, and fungus could be affected by the study quality, which is significantly associated with the reliability of abstracted data.

This study had several limitations. First, all the included studies had a retrospective design and uncontrolled selection. Hence, recall biases were inevitable. Second, all the included studies were of relatively low or moderate quality, and the summary results were restricted for clinical application. Third, the analysis of drug resistance was not available, which needs further study. Fourth, pathogen distribution might differ by region and pneumonia subtypes, whereas the stratified analyses based on these factors were not performed. Fifth, all of the included studies were performed in China, and the recommendation of results in our study to other countries was restricted. Finally, the analysis was based on published articles. Hence, publication bias was inevitable.

In summary, the findings of this study indicated that gram-negative bacilli were the most common bacterial infection in elderly patients with pneumonia, and the most common types of gram-negative bacilli were *P. aeruginosa* and *K. pneumoniae*. Moreover, *S. aureus, S. hemolyticus*, and *S. pneumoniae* were the most common gram-positive cocci in elderly patients with pneumonia. The most common fungus in elderly patients with pneumonia was *C. albicans*. Appropriate antibiotics should be applied based on the microbial surveillance data of each hospital.

## Data Availability Statement

The original contributions presented in the study are included in the article/Supplementary Material, further inquiries can be directed to the corresponding authors.

## Author Contributions

LC substantially contributed to the conception, acquisition, analysis, and interpretation of data and drafted the manuscript for important content. HH contributed to design and critically revised the manuscript for important intellectual content. XC contributed to the acquisition of data and all authors gave final approval.

## Conflict of Interest

The authors declare that the research was conducted in the absence of any commercial or financial relationships that could be construed as a potential conflict of interest.

## Publisher's Note

All claims expressed in this article are solely those of the authors and do not necessarily represent those of their affiliated organizations, or those of the publisher, the editors and the reviewers. Any product that may be evaluated in this article, or claim that may be made by its manufacturer, is not guaranteed or endorsed by the publisher.
