# Supplementary material for: Distribution of Pathogens in Elderly Chinese Patients With Pneumonia: A Systematic Review and Meta-Analysis
Source: Front Med (Lausanne). 2021 Jul 26;8:584066. doi: 10.3389/fmed.2021.584066 (PMC8350134; doi:10.3389/fmed.2021.584066)
Supplement: Supplementary file 1 [file Data_Sheet_1.doc]

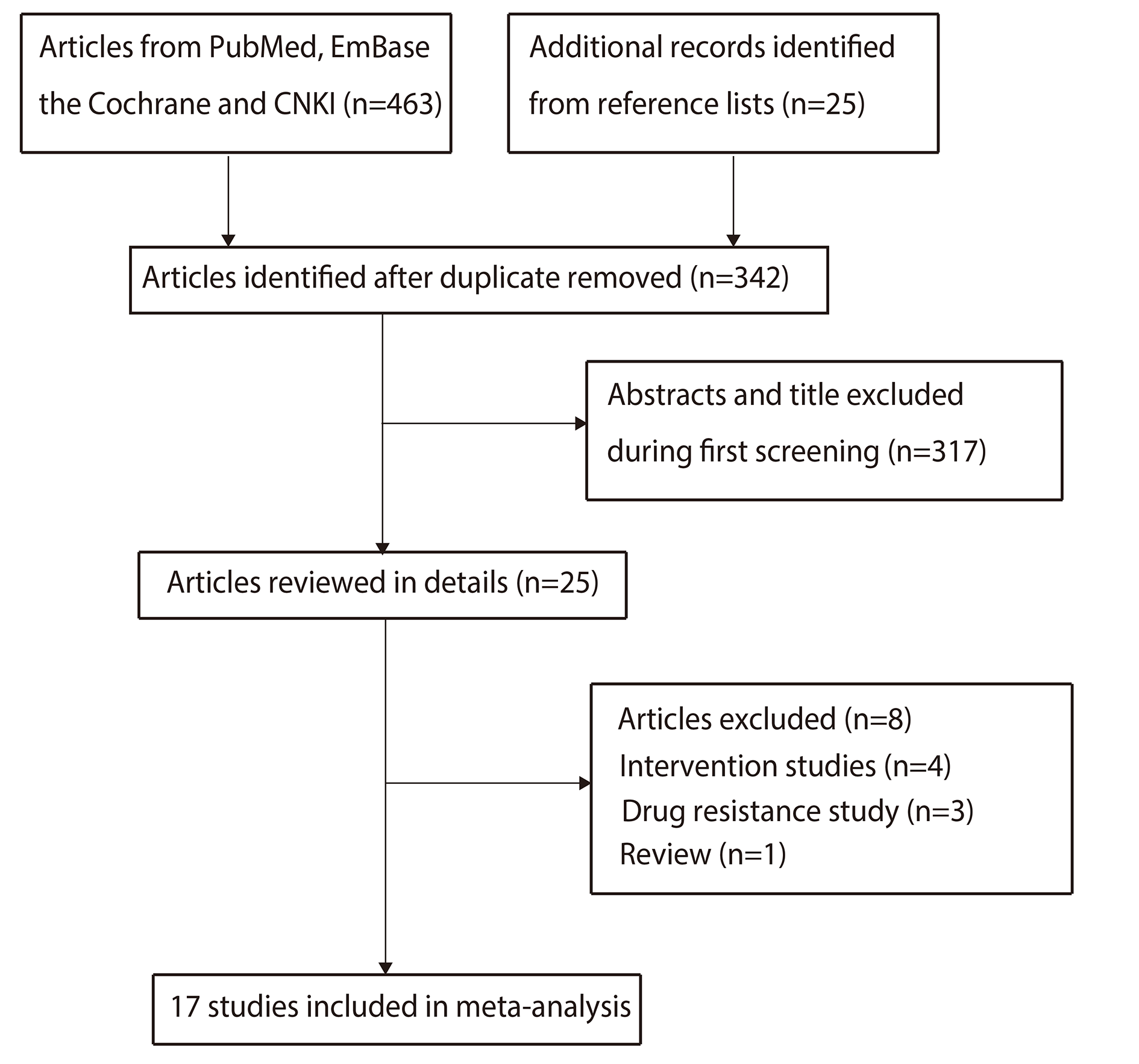


Additional Figure S1. Flow diagram of the literature search and study selection process.


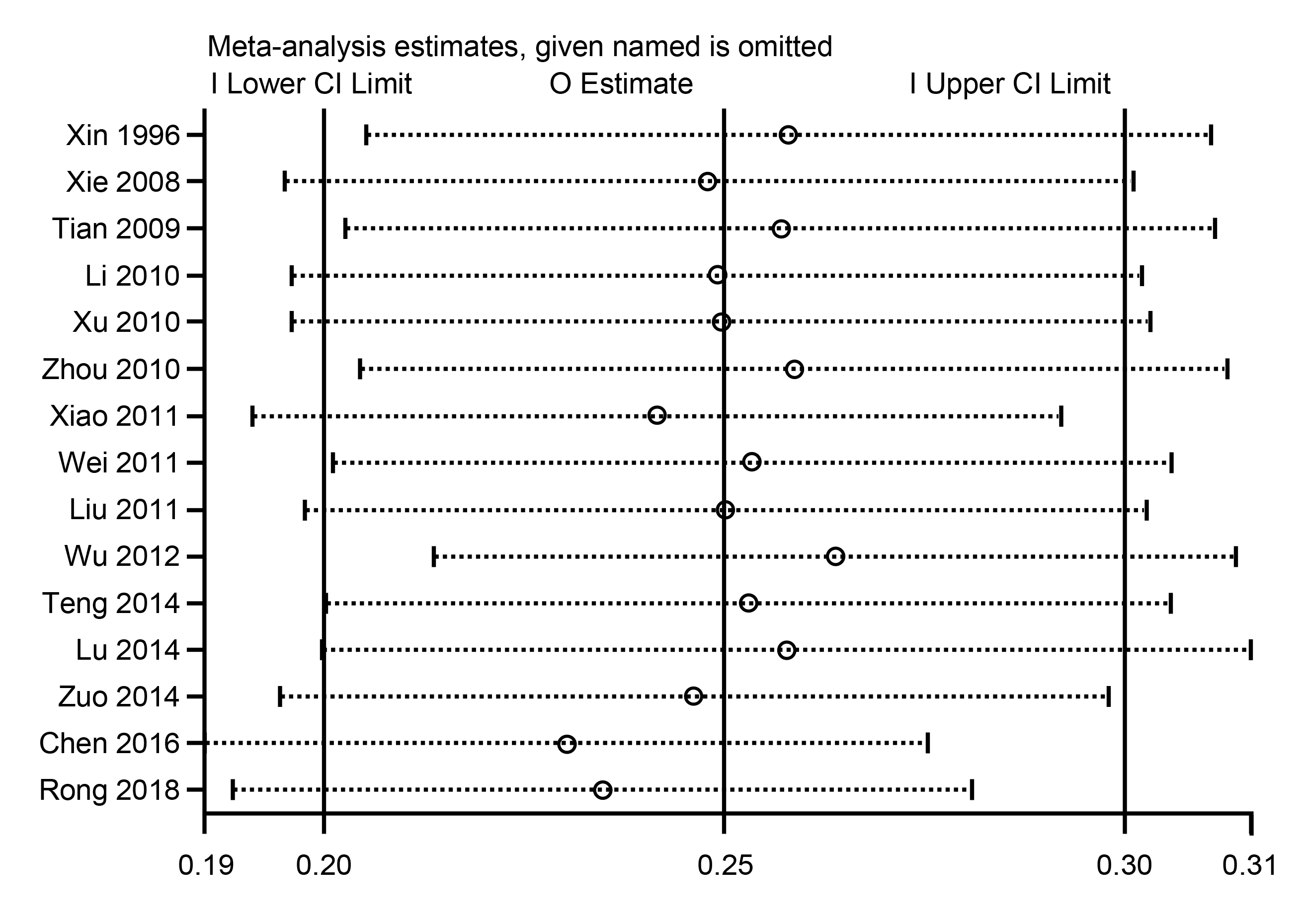


Additional Figure S2. Sensitivity analysis for gram-positive cocci.


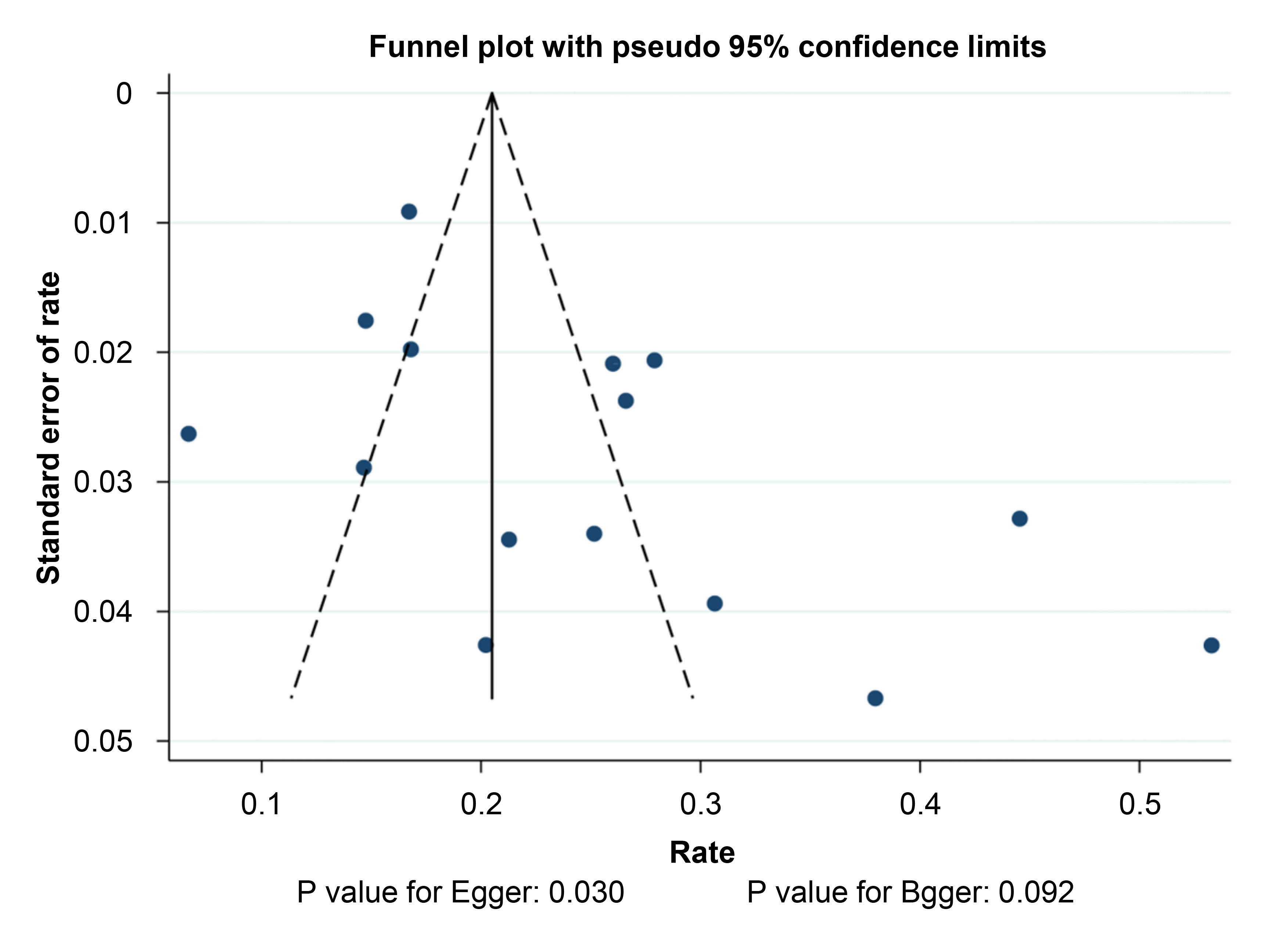


Additional Figure S3. Funnel plot for gram-positive cocci.


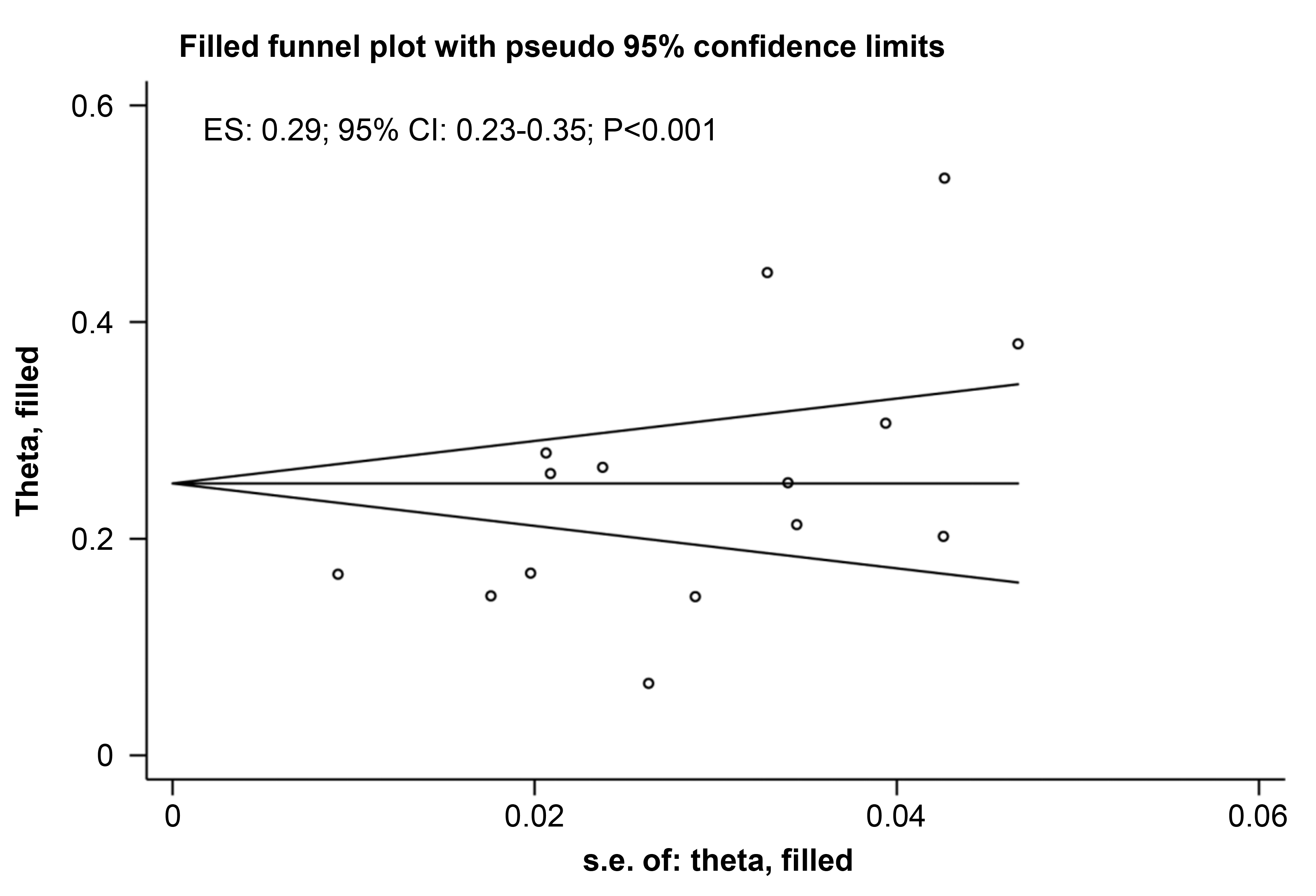


Additional Figure S4. Trim and fill for gram-positive cocci.


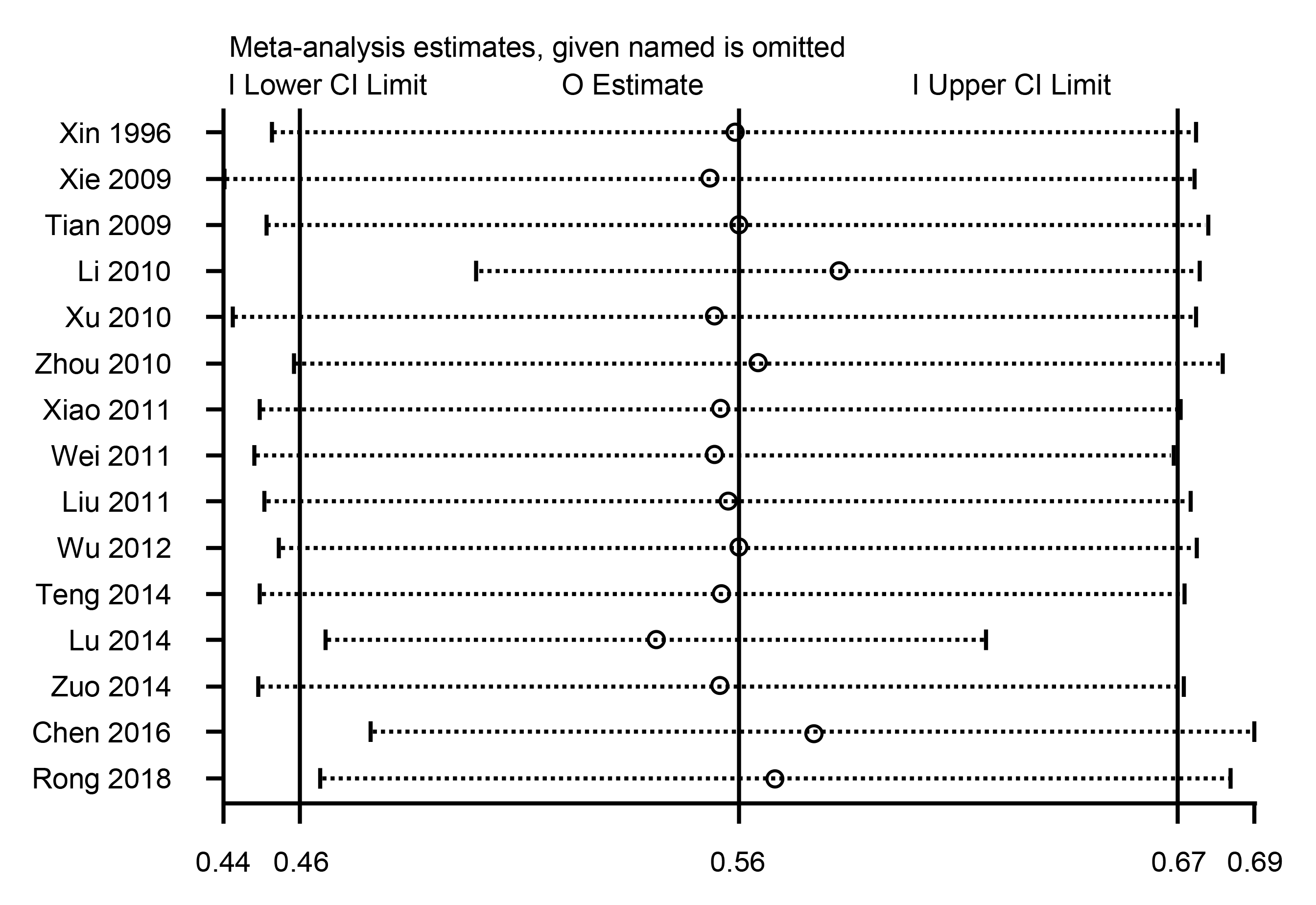


Additional Figure S5. Sensitivity analysis for gram-negative bacilli.


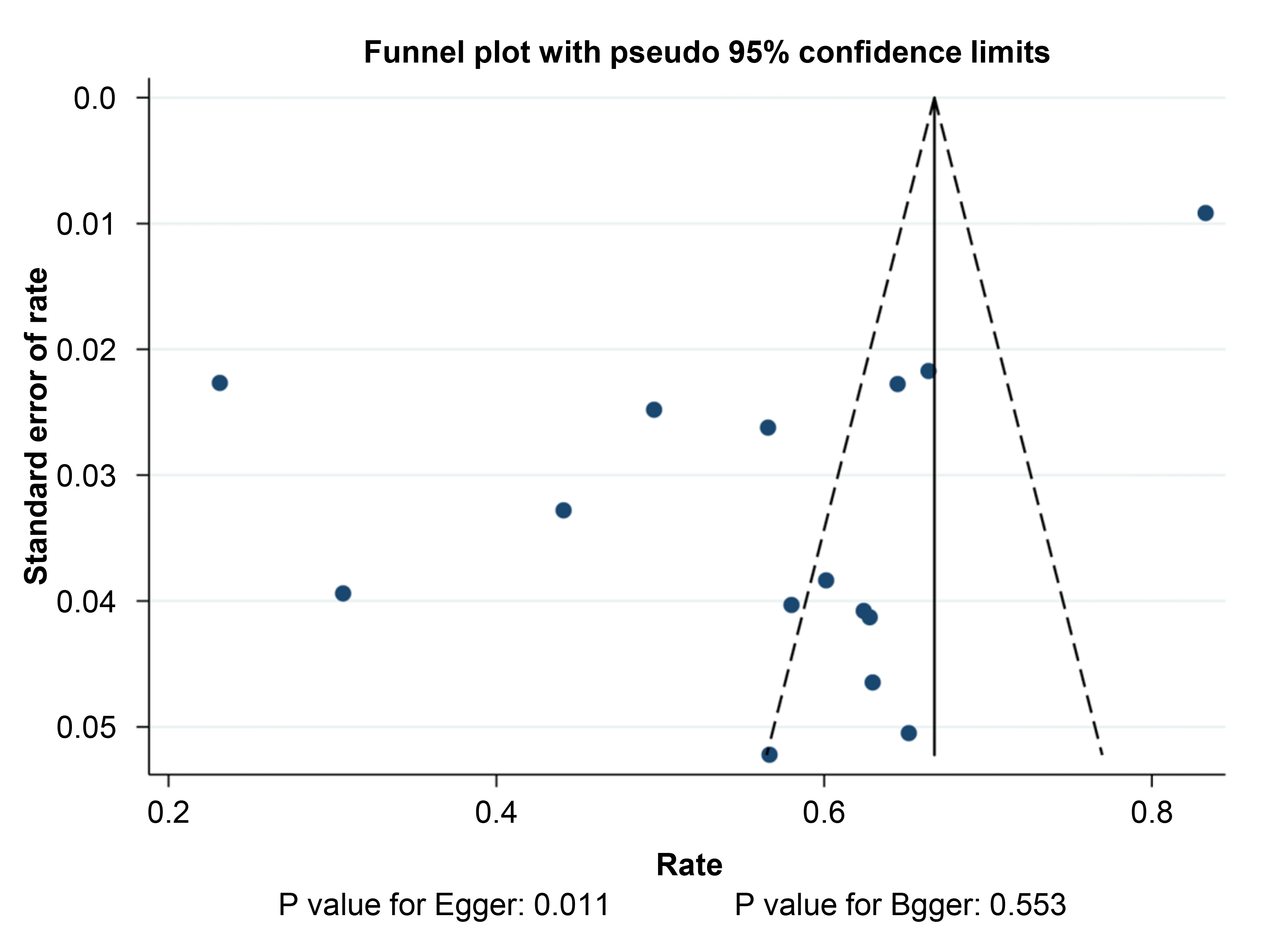


Additional Figure S6. Funnel plot for gram-negative bacilli.


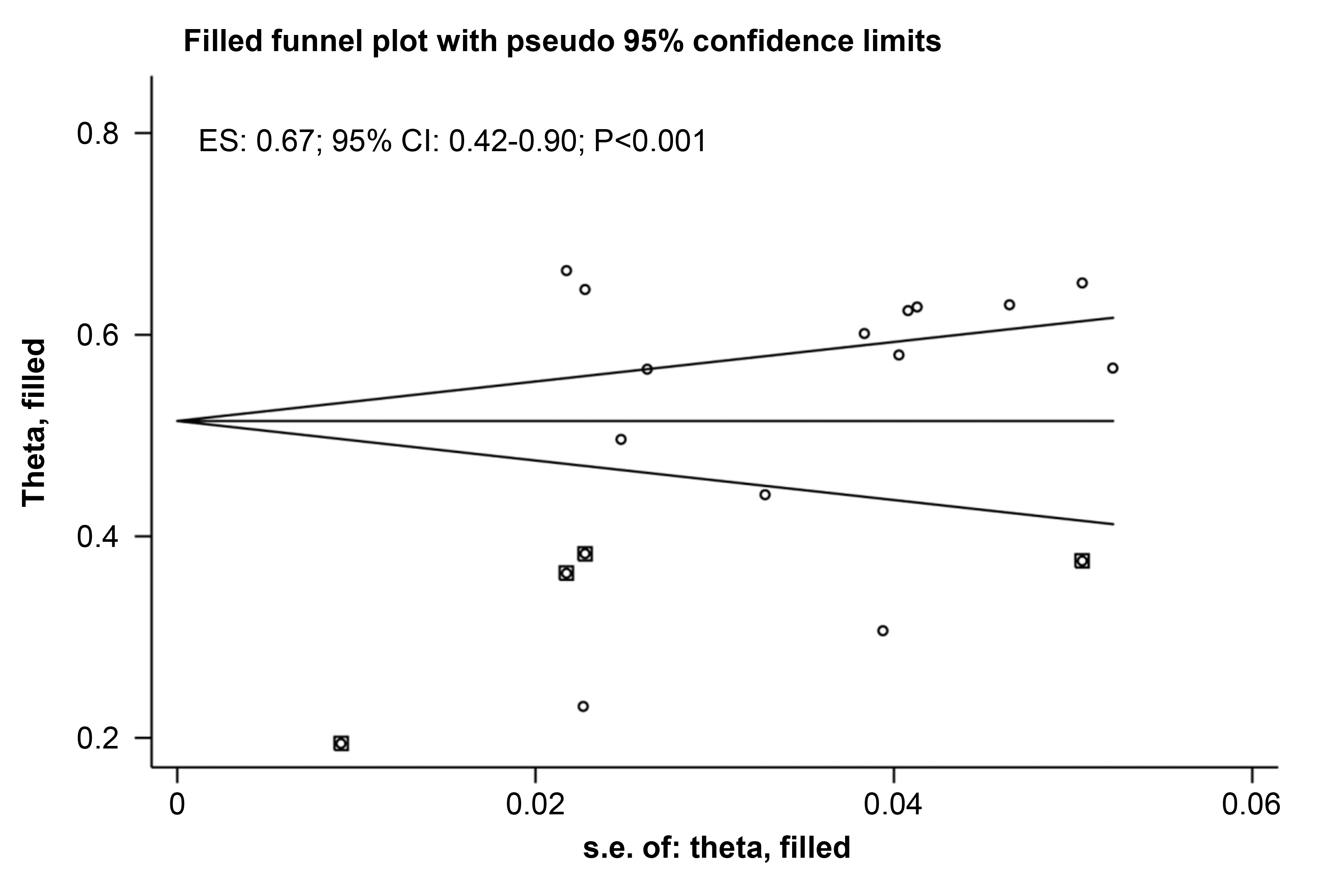


Additional Figure S7. Trim and fill for gram-negative bacilli.


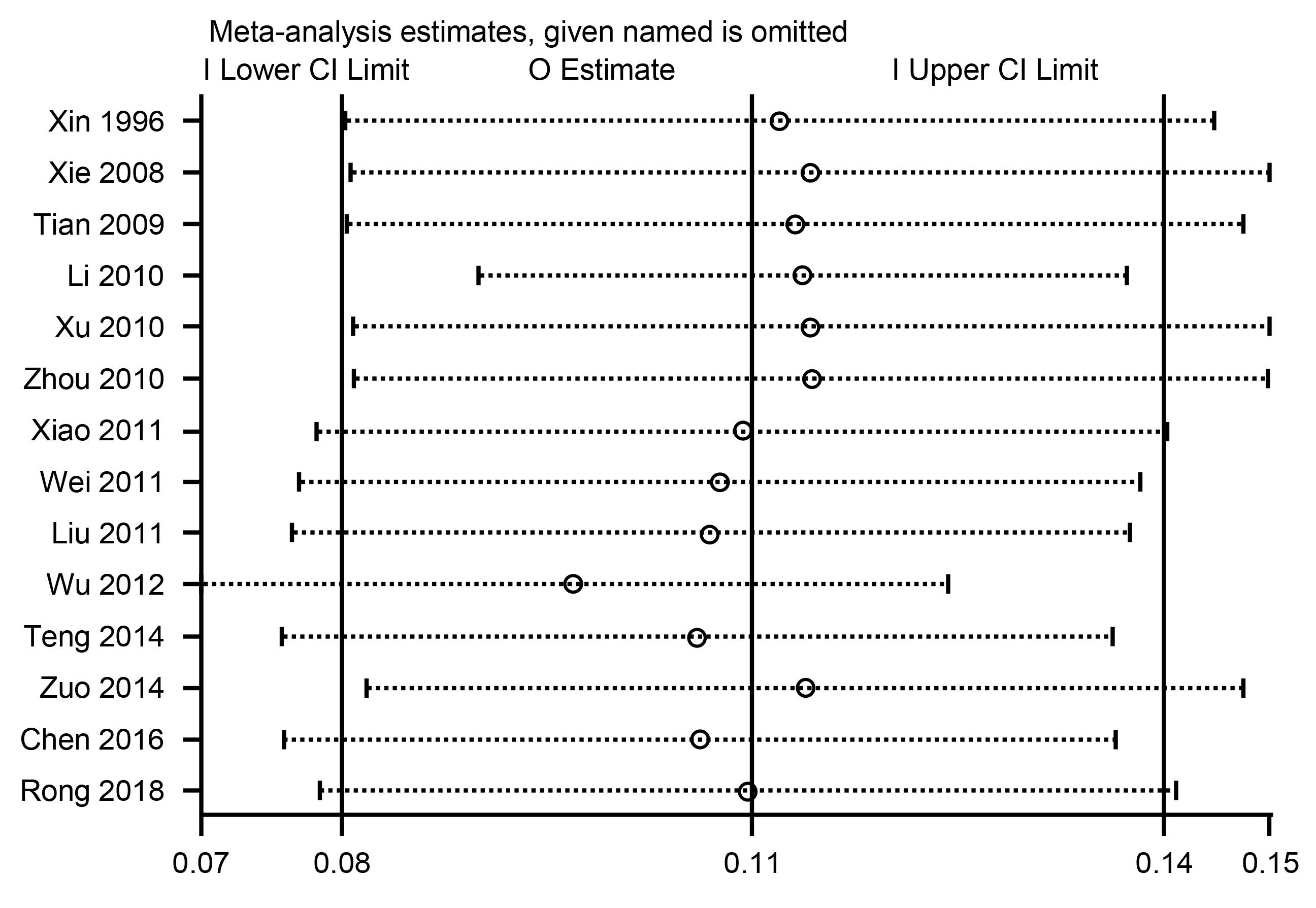


Additional Figure S8. Sensitivity analysis for fungus.


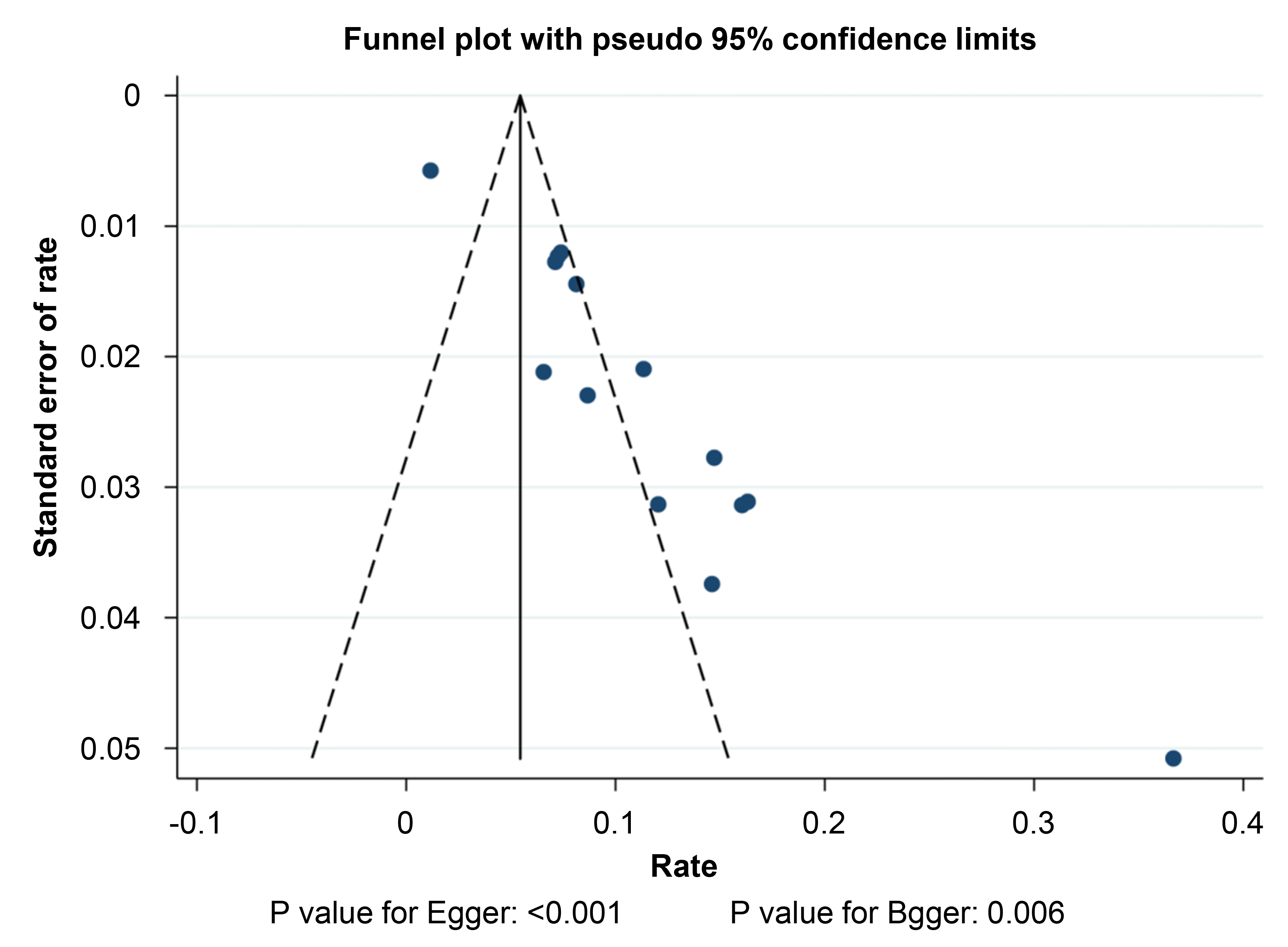


Additional Figure S9. Funnel plot for fungus.


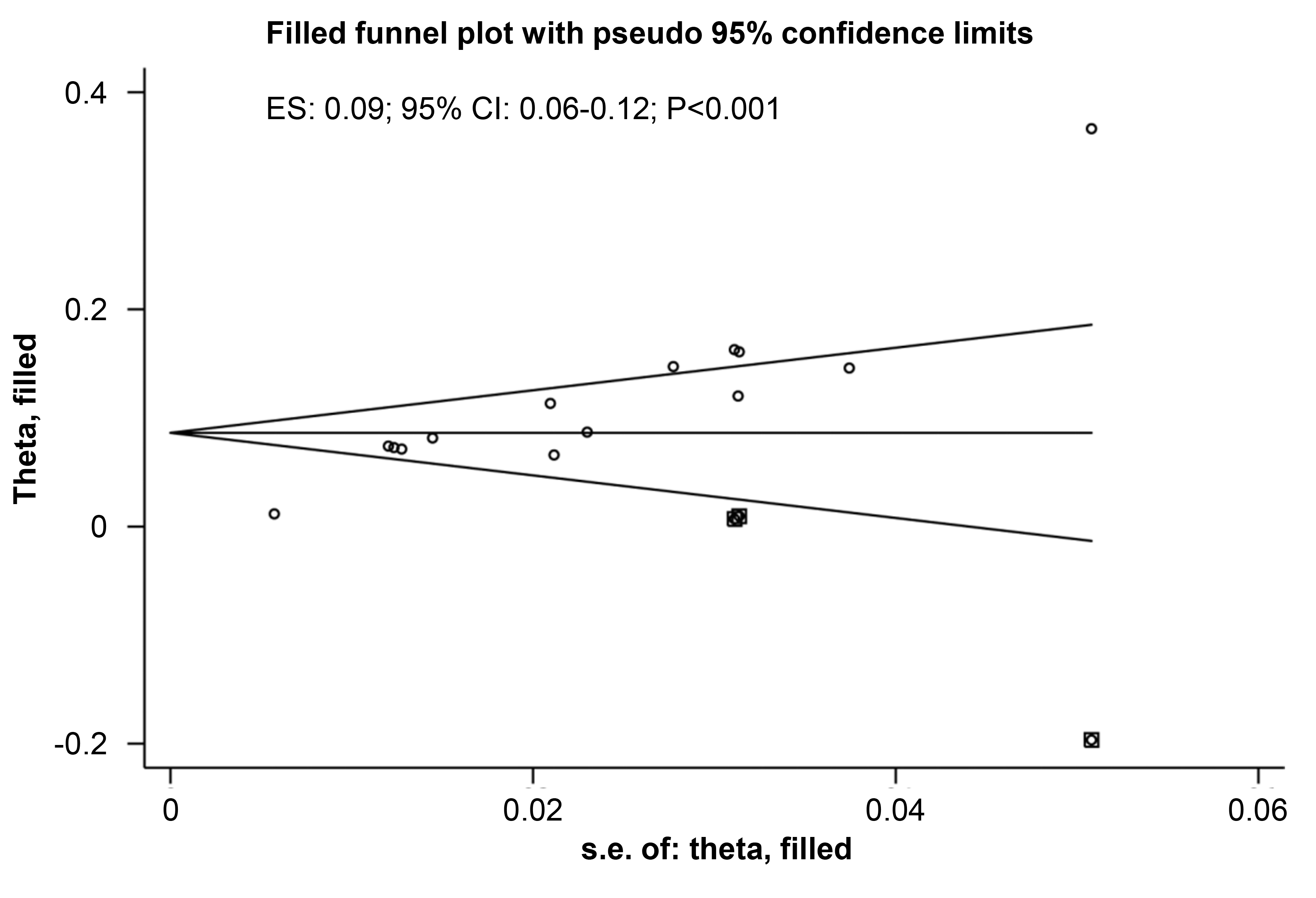


Additional Figure S10. Trim and fill for fungus.
