# Supplementary material for: Distribution of Pathogens in Elderly Chinese Patients With Pneumonia: A Systematic Review and Meta-Analysis
Source: Front Med (Lausanne). 2021 Jul 26;8:584066. doi: 10.3389/fmed.2021.584066 (PMC8350134; doi:10.3389/fmed.2021.584066)
Supplement: Supplementary file 2 [file Data_Sheet_2.docx]

Table S1. Baseline characteristics of the studies included in the systematic review and meta-analysis

| Study | Study period | Region | Study design | Sample size | Age (mean age) | Number of men/women | Pneumonia subtypes | Pathogen analysis | Study quality |
| --- | --- | --- | --- | --- | --- | --- | --- | --- | --- |
| Xin 1996 (Xin C et al., 1996) | 1992-1996 | Beijing | Retrospective | 150 | > 60.0 | 84/66 (56%) | NA | Sputum culture analysis | 4 |
| Xie 2008 (Xie X et al., 2008) | 2000-2007 | Beijing | Retrospective | 473 | > 60.0 | 385/88 (81.4%) | NA | Sputum culture analysis | 5 |
| Tian 2009 (Tian & Shang, 2009) | 2005-2007 | Shandong | Retrospective | 357 | 60.0-89.0 (74.0) | 212/145 (59.4%) | CAP and HAP | Sputum culture analysis | 5 |
| Li 2010 (Li J & X., 2010) | 2007-2009 | Guangdong | Retrospective | 346 | 60.0-97.0 (81.0) | 239/107 (69.1%) | CAP and HAP | Sputum culture analysis | 4 |
| Xu 2010 (Xu X et al., 2010) | 2003-2010 | Jiangsu | Retrospective | 442 | 61.0-95.0 | 365/77 (82.6%) | NA | Sputum culture analysis | 5 |
| Zhou 2010 (X., 2010) | 2007-2009 | Jiangsu | Retrospective | 407 | 60.0-89.0 (74.0) | 237/170 (58.2%) | CAP and HAP | Sputum culture analysis | 5 |
| Xiao 2011 (D., 2011) | 2007-2010 | Hunan | Retrospective | 108 | 75.0 | 62/46 (57.4%) | NA | Sputum culture analysis | 4 |
| Wei 2011 (Z., 2011) | 2005-2010 | Guangxi | Retrospective | 89 | 60.0-89.0 | 25/61 (28.1%) | NA | Sputum culture analysis | 4 |
| Liu 2011 (Liu et al., 2011) | 2007-2009 | Beijing | Retrospective | 146 | 60.0-91.0 (76.0) | 105/41 (71.9%) | CAP and HAP | Sputum culture analysis | 5 |
| Wu 2012 (Wu C et al., 2012) | 2009-2011 | Guangdong | Retrospective | 300 | NA | NA | NA | Sputum culture analysis | 3 |
| Zheng 2012 (Zheng Y & L., 2012) | 2010 | Hubei | Retrospective | 277 | NA | NA | NA | Sputum culture analysis | 4 |
| Zhang 2012 (Zhang G & X., 2012) | 2007-2011 | Liaoning | Retrospective | 326 | NA | NA | NA | Sputum culture analysis | 3 |
| Teng 2014 (L., 2014) | 2008-2010 | Hunan | Retrospective | 125 | 75.2 | 79/46 (63.2%) | NA | Sputum culture analysis | 4 |
| Lu 2014 (Lu Y et al.) | 2011-2012 | Zhejiang | Retrospective | 1636 | 60.0-92.0 (75.23) | 905/731 (55.3%) | CAP and HAP | Sputum culture analysis | 5 |
| Zuo 2014 (Zuo, 2014) | 2011-2013 | Sichuan | Retrospective | 200 | 60.0-92.0 (77.6) | 134/66 (67.0%) | CAP | Sputum culture analysis | 4 |
| Chen 2016 (Chen et al., 2016) | 2014-2016 | Guangdong | Retrospective | 118 | > 60.0 (75.11) | 68/50 (57.6%) | NA | Sputum culture analysis | 4 |
| Rong 2018 (Rong S & X., 2018) | 2010-2015 | Qinghai | Retrospective | 229 | 60.0-77.0 (68.4) | 114/115 (49.8%) | NA | Sputum culture analysis | 4 |

*CAP: community-acquired pneumonia; HAP: hospital acquired pneumonia; NA: not available

**References**

Chen, B., Zhao, W., & Pharmacy, D. Analysis on Distribution of Pathogen and Application Rationality of Elderly Patents with Pneumonia. 2016;

D., X. Analysis of pathogenic bacteria and drug resistance in 108 elderly patients with pneumonia. Chin Modern Med 2011; 18:74-75.

L., T. Observation on the distribution and drug resistance of pathogenic bacteria in pneumonia patients. Chin Foreign J Med Res 2014; 12:147-148.

Li J, & X., P. Distribution and sensitivity analysis of pathogenic bacteria in 3 46 cases of senile pneumonia. Chin J Rural Med 2010; 17:58-59.

Liu, Z. D., Juan-Li, L., Zhou, G. J., Jia, C., & Liu, Y. Distribution of pathogens causing pneumonia in senile patients and drug resistance. Chinese Journal of Nosocomiology 2011;

Lu Y, Zheng H, & Qiu Y. Analysis of pathogenic bacteria and drug resistance of senile pneumonia in island area. Zhejiang Med J 2014 36:231-233.

Rong S, & X., Z. The Pathogen Distribution and its Effect on TGF β-Smad Signaling Pathway Proteins in Patients with Elderly Pneumonia. Chin High Altit Med Biol 2018; 39:39-43.

Tian, H., & Shang, X. M. Pathogens in Senile Pneumonia Patients:Their Distribution and Resistance Analysis. Chinese Journal of Nosocomiology 2009;

Wu C, Tan S, & Li D. Pathogens distribution and analysis of drug resistance in senile patients with pneumonia. Inner Mongolia J Trad Chin Med 2012; 2:65-66.

X., Z. Distribution and drug resistance of pathogenic bacteria in elderly patients with pneumonia. Occup and Health 2010; 26:2811-2813.

Xie X, Tang H, & Q., W. Analysis of pathogenic bacteria distribution and antimicrobial application in elderly patients with pneumonia. Pharm J Chin PLA 2008; 24: 462-463.

Xin C, Feng J, & Y., L. Analysis of pathogenic bacteria and drug susceptibility in 150 elderly patients with pneumonia. Beijing Military Med J 1996; 8:49-50.

Xu X, Huang Y, & D., R. Analysis of common pathogenic bacteria and drug resistance of senile pneumonia. J Nantong Univers 2010; 30:290-292.

Z., W. Distribution and drug susceptibility analysis of pathogenic bacteria in elderly patients with hypothyroidism pneumonia. Guangxi Med J 2011; 33:1496-1497.

Zhang G, & X., G. Distribution characteristics and drug resistance analysis of pathogenic bacteria in sputum culture of pneumonia in the aged and children. Chin Prac Med 2012; 7:180-182.

Zheng Y, & L., C. Analysis of pathogenic bacteria distribution and drug resistance in elderly patients with pneumonia. Chin J Nosocomiol 2012; 22:858-859.

Zuo, T. H. Pathogens distribution and drug resistance analysis and countermeasures of elderly patients with pneumonia. Journal of Clinical Pulmonary Medicine 2014;

Table S2. Subgroup analyses for the incidence of gram-positive cocci, gram-negative bacilli and fungus

| Outcomes | Groups | Incidence and 95% CI | p-value | Heterogeneity (%) | | p-value for Heterogeneity | | | p-value between subgroups |
| --- | --- | --- | --- | --- | --- | --- | --- | --- | --- |
| Gram-positive cocci | Mean age (years) |  | | | | | | | |
|  | > 75.0 | 0.30 (0.21-0.38) | <0.001 | 94.3 | | <0.001 | | | 0.720 |
|  | < 75.0 | 0.25 (0.10-0.40) | 0.001 | 97.0 | | <0.001 | | |  |
|  | NA | 0.19 (0.11-0.27) | <0.001 | 929.2 | | <0.001 | | |  |
|  | Perentage male (%) |  | | | | | | | |
|  | > 70.0 | 0.27 (0.24-0.29) | <0.001 | 0.0 | | 0.720 | | | <0.001 |
|  | < 70.0 | 0.27 (0.21-0.33) | <0.001 | 94.3 | | <0.001 | | |  |
|  | NA | 0.07 (0.02-0.12) | 0.011 | - | | - | | |  |
|  | Study quality |  | | | | | | | |
|  | 5 | 0.21 (0.17-0.25) | <0.001 | 89.2 | | <0.001 | | | <0.001 |
|  | 4 or 3 | 0.28 (0.19-0.38) | <0.001 | 94.8 | | <0.001 | | |  |
| Gram-negative bacilli | Mean age (years) |  | | | | | | |  |
|  | > 75.0 | 0.55 (0.32-0.78) | <0.001 | 99.2 | | <0.001 | | | <0.001 |
|  | < 75.0 | 0.50 (0.44-0.57) | <0.001 | 78.4 | | 0.010 | | |  |
|  | NA | 0.64 (0.60-0.67) | <0.001 | 27.2 | | 0.241 | | |  |
|  | Perentage male (%) |  | | | | | | | |
|  | > 70.0 | 0.65 (0.62-0.68) | <0.001 | 1.7 | | 0.361 | | | 0.039 |
|  | < 70.0 | 0.54 (0.39-0.69) | <0.001 | 98.9 | | <0.001 | | |  |
|  | NA | 0.57 (0.46-0.67) | <0.001 | - | | - | | |  |
|  | Study quality |  | | | | | | | |
|  | 5 | 0.64 (0.51-0.76) | <0.001 | 98.3 | | <0.001 | | <0.001 | |
|  | 4 or 3 | 0.52 (0.39-0.64) | <0.001 | 95.8 | | <0.001 | |  |  |
| Fungus | Mean age (years) |  | | | | | |  |  |
|  | > 75.0 | 0.11 (0.04-0.17) | 0.001 | 93.3 | | <0.001 | | <0.001 | |
|  | < 75.0 | 0.08 (0.06-0.11) | <0.001 | 32.8 | | 0.226 | |  |  |
|  | NA | 0.13 (0.07-0.18) | <0.001 | 88.7 | | <0.001 | |  |  |
|  | Perentage male (%) |  | | | | | | | |
|  | > 70.0 | 0.09 (0.06-0.12) | <0.001 | 69.2 | | 0.039 | <0.001 | | |
|  | < 70.0 | 0.10 (0.06-0.13) | <0.001 | 91.0 | | <0.001 |  |  |  |
|  | NA | 0.37 (0.27-0.47) | <0.001 | - | | - |  |  |  |
|  | Study quality |  | | | | | | | |
|  | 5 | 0.08 (0.06-0.10) | <0.001 | 41.8 | 0.143 | | <0.001 | | |
|  | 4 or 3 | 0.13 (0.07-0.19) | <0.001 | 93.8 | <0.001 | |  |  |  |
